# Biological Robots: Perspectives on an Emerging Interdisciplinary Field

**DOI:** 10.1089/soro.2022.0142

**Published:** 2023-08-09

**Authors:** Douglas Blackiston, Sam Kriegman, Josh Bongard, Michael Levin

**Affiliations:** ^1^ Department of Biology, Allen Discovery Center at Tufts University, Medford, Massachusetts, USA.; ^2^ Wyss Institute for Biologically Inspired Engineering, Harvard University, Boston, Massachusetts, USA.; ^3^ Institute for Computationally Designed Organisms, Massachusetts and Vermont, USA.; ^4^ Center for Robotics and Biosystems, Northwestern University, Evanston, Illinois, USA.; ^5^ Center for Synthetic Biology, Northwestern University, Evanston, Illinois, USA.; ^6^ Department of Computer Science, University of Vermont, Burlington, Vermont, USA.

**Keywords:** embryo, developmental biology, robot, xenobot, computer science, synthetic bioengineering, biorobot, animal cap

## Abstract

Advances in science and engineering often reveal the limitations of classical approaches initially used to understand, predict, and control phenomena. With progress, conceptual categories must often be re-evaluated to better track recently discovered invariants across disciplines. It is essential to refine frameworks and resolve conflicting boundaries between disciplines such that they better facilitate, not restrict, experimental approaches and capabilities. In this essay, we address specific questions and critiques which have arisen in response to our research program, which lies at the intersection of developmental biology, computer science, and robotics. In the context of biological machines and robots, we explore changes across concepts and previously distinct fields that are driven by recent advances in materials, information, and life sciences. Herein, each author provides their own perspective on the subject, framed by their own disciplinary training. We argue that as with computation, certain aspects of developmental biology and robotics are not tied to specific materials; rather, the consilience of these fields can help to shed light on issues of multiscale control, self-assembly, and relationships between form and function. We hope new fields can emerge as boundaries arising from technological limitations are overcome, furthering practical applications from regenerative medicine to useful synthetic living machines.

## Introduction

Multidisciplinary research programs have the potential to generate important advances both within and between established fields; the integration of existing ideas and techniques among researchers with diverse backgrounds often leads to new approaches and new questions. This can be seen in biological robotics, where living materials are being used to build new kinds of robotic devices. Progress in this nascent field will depend on the development of a shared lexicon, as many terms central to biology and robotics lack operational definitions and can have multiple, sometimes incompatible, interpretations when referenced by different fields. For example, what at its essence is a robot? Machine? Organoid? Organism? How these terms are used is contingent upon many factors, including disciplinary training, journal readership, reviewers, classical terminology, and the target audience.

Debates surrounding biological robotics and its nomenclature can be observed in articles, at conferences, in the popular press, and increasingly on social media. Here, we provide individual perspectives on specific questions and critiques raised across disciplines with respect to our recent work on biological robots. This essay is not meant to serve as a review of biological robots, biorobotics, or artificial intelligence (AI)-designed biology at large, but rather to provide our perspectives on specific key issues that have been raised with regard to our research program and related forthcoming technologies. The individual viewpoints of our interdisciplinary team are sometimes nonoverlapping, yet are integrated toward the overarching goal of improving frameworks for future research, and identifying areas in which dichotomous thinking creates artificial boundaries in understanding.

## Dovetailing Developmental Biology and Robotics (Blackiston Commentary)

Biorobotics and materials synthetic biology are relatively young disciplines, with both witnessing a surge in progress across the preceding decade.^1–6^ Approaches, model systems, and goals are myriad, combining elements of bioengineering, stem cell biology, molecular biology, computer science, engineering, neuroscience, and robotics. My work lies at the intersection of these fascinating disciplines, building self-motile biological machines from the ground up from both user and AI-inspired anatomies, engineered for a specific purpose.^7–9^ While these results have engendered broad support from the scientific community and general public, several pointed critiques have been raised by members of the developmental biology community across social media platforms,^10^ which are important when framing the work both within the discipline and to those outside the field. As several of these points are valid, the current author offers here their view on the respective topics, which does not necessarily reflect the view of the other co-authors.

The most common point of discussion is that the system is not a robot nor engineered, it is what developmental biologists traditionally define as an animal cap,^11–17^ a region of developing frog embryo that gives rise to epidermis and neural tissue in the native system. This position has merit, yet indicates a narrow view of the work and fails to acknowledge the diverse biological machines produced by the design pipeline. The initial research study was comparable to several biohybrid robots, which use a combination of synthetic scaffolds and cardiac muscle-based actuation to generate forward locomotion.^18–22^ Using these same muscle-based actuators, my designs replaced the synthetic scaffolds with living materials, creating a combination of modified animal cap- and muscle-derived tissues.^8^

**FIG. 1.**
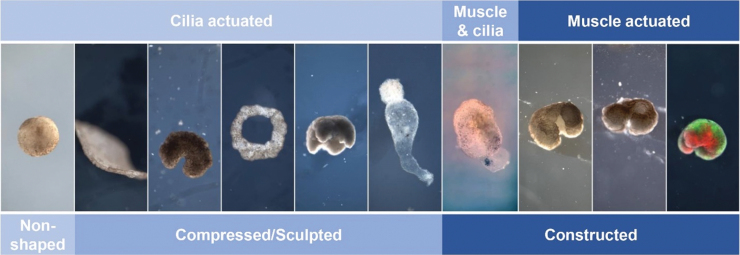
Sample of designs produced in the research program. Actuation can be achieved through motile cilia generated flow, contractile muscle tissue, or a combination of the two. Morphology can be generated via compression, sculpting, or by layering specific tissue types during the construction process (*far right panel*, *red* indicates muscle, *green* indicates epidermis). 5th panel modified and reprinted with permission from ref. [8], National Academy of Sciences of the United States of America.

In the follow-up work, the team explored the use of motile cilia-based actuation, small hairlike structures naturally present on the surface of tadpole epidermis.^7,9^ The simplest of these designs are indeed spherical animal caps, a point acknowledged in the article, which were used to quantify metrics of velocity and life span of ciliated tissues and further advance several computational models. However, the author's view is that this approach represents an advance in materials synthetic biology rather than developmental biology. Tissues and cells were leveraged as materials to create a sliding scale of design, from fully muscle actuated to fully cilia actuated, as well as combinations of the two, and from non-shaped spheroids to carefully designed anatomies with multiple tissue types placed at specific locations (Fig. 1).

Related to this first point is the idea that the locomotive behavior of animal cap-derived tissue is a known phenomenon, a position with which I am in agreement. The fact that ciliated tissues generate sufficient flow to create motion has been reported in the literature for decades,^23–25^ and the current author would not claim to have discovered something new or unexpected in the system. To the contrary, the results indicate that the cilia and muscle actuators behave like windup toys in a predictable and stereotyped manner, a feature which was leveraged with modeling to simulate how individual, and groups, of biological machines will behave *in vitro*.

It is precisely through manipulation of these known and stereotyped behaviors that we were able to generate kinematic self-replication,^9^ a fragile process requiring careful titration of several input variables from the investigator. Thus, the innovation comes not from novel developmental insight, but rather using these known systems as living materials, enhancing, or changing movement and behavior through anatomical design by placing actuators at specific locations, or by manually layering tissues into novel configurations. A future goal remains better control of the design process to generate motion and behaviors, which would not be predicted in traditional animal caps.

Similarly, there has been controversy when using the name xenobots to describe the biological machines, as it represents a rebranding of the established animal cap nomenclature. This author avoids the term xenobot as it is a nontechnical descriptor, and one that was not used in the first article, nor the third following community feedback. However, an umbrella term remains necessary to capture the full design space of the research program, which includes cilia and muscle-based actuation, various tissue geometries, sizes, and in the future, cells derived from diverse taxa. Internally, team members disagree regarding the correct vocabulary to describe the system. These terms include: reconfigurable organisms, computer-designed organisms, biobots, xenobots, engineered living systems, biological robots, and biomachines, with the latter being the present author's preferred term as it captures the full range of design space.

Additionally, this term should only be applied to the system where motility is rationally designed or used to complete human specified work, and an unmodified animal cap, or the differentiated mucociliary organoid,^24,26–29^ should be labeled as such where applicable. Finally, while reconfigurable organism is the specific nomenclature used in the articles, this term is also problematic as biologists currently have no operational definition of the word organism,^30,31^ with some arguing the term is without technical meaning (the current author does not consider the designs organisms by any definition).^32^

There has also been dispute when using the terms “machine” and “robot” to describe the designs. Here, I must respectfully disagree with some members of the developmental biology community. Across this multidisciplinary research process, the author has come to appreciate that robots are defined by their rational design, autonomy, and ability to do human specified work, rather than by the fabrication materials. Indeed, many robots are constructed from nonintuitive components including cardiac powered biohybrid designs,^20,22,33–35^ pinecone and oat seed robots driven by hygromorphic actuators,^36,37^ liquid droplets,^38–41^ and a light-driven *Caenorhabditis elegans* RoboWorm,^42^ to name but a few.

In this framing, there is little difference between a biohybrid robot constructed from a synthetic soft-scaffold and muscle tissue and one constructed from a living soft-scaffold and muscle tissue. Both are designed for a human purpose, fabricated, and evaluated for performance, and both function through predictable open-loop control systems. As progress continues toward allowing our designs to sense and respond to their environment, their definition will begin to skew from biomachine toward biobot proper. These principles and continued exposure to the robotics field have been rewarding and inspiring for the author, who hopes members of other communities will likewise have an open mind when discussing the discipline.

Beyond the immediate science, an important point of discourse surrounding this research relates to the larger issue of science communication, in its many forms and venues.^43^ How, and where, should one promote one's own findings to both the scientific community and general public in an increasingly digital age? Our current research has been fortunate to receive significant visibility from the popular press, in both written and video formats. Engaging with these venues remains up to the investigator and there exists potential for both scientific benefit and risk with either course of action. Sensationalism, especially in headlines, drives traffic and revenue for periodicals, and a resistance to commentary with the popular press removes an important check to accurately reporting one's results.

Specifically, several periodicals have implied, or state definitively, that the system contains various degrees of cognition, or that the cells spontaneously generate novel functionality and behaviors rather than being designed by an investigator. However, as the scientist who envisioned and constructed all of the *in vitro* designs to date, the data do not support these speculations. Correcting these statements *post hoc* becomes challenging as the articles are often managed by a website publisher, meaning the original journalist no longer has editorial oversight of the piece. In other cases, publishers have been unwilling to address concerns, and the present author has removed their name from several articles, which misrepresent the research findings.

This is not a solution, however, as the incorrect message remains public facing in perpetuity. Further, close engagement with popular press represents a potential conflict of interest, as the investigator is both producing and selling the product, effectively creating a non-reliable narrator and eroding trust in the discipline at large.^44^ While the current author maintains caution when engaging with the press, it is also a reality that these venues generate value as privatized funding sources become increasingly common.

Finally, peer review, both during publication and through subsequent dissemination of the work remains essential to the research process. Presently, we have witnessed this review moving from the realm of grants, articles, and conferences into the digital world in the form of social media. The benefits here are many; investigators can rapidly reach a large community when sharing ideas, troubleshooting methods, creating a professional network, and providing scientific critique. However, there is also danger when scientific critique is built on an emotional response toward an individual rather than rational analysis of a result.^45^

As a community, we should continue to assume the best in our colleagues. Should a particular result or finding appear misplaced, one's goal should always be to improve the science rather than tear down an individual, and mentorship remains essential to members at all levels of academic positions. However, it is also necessary that scientists separate speculation from data, and we must absolutely hold our colleagues to the highest standards if we are to maintain public trust in scientific results. The current author has had to learn scientific communication on the fly, falling short on several occasions, and continues to appreciate the community feedback as he develops in this space.

## From Strange Feet to Strange Machines (Kriegman Commentary)

In Jewish folklore and Greek mythology, it's relatively easy to create an intelligent robot: Simply combine mud (Golem) or sculpt ivory (Galatea) into the right shape. Without divine intervention, it's much harder to bring inanimate objects to life. Transforming containers of fluids and elastomers into a wiggling soft robot requires careful design and precise manufacturing.^46^ To get such a robot to do something else besides wiggling in place is exceedingly difficult.

So, why not start with living materials instead? In the “xenobots” project,^7–9^ that's exactly what we did. Creating a self-powered, self-driving, self-repairing, self-replicating robot (Table 1) was not trivial—doing so required careful planning, design, and construction—but it was, in essence, as simple as combining and sculpting the right material into the right shape. In our case, the right material was *Xenopus* cells and the right shape was one that, in computer simulations, maximized the likelihood of generating an interesting nonrandom behavior, such as forward locomotion.

**Table 1.** Description of Terms Used by Each Investigator

Term	Author	Definition
Xenobot	Blackiston	Avoids usage of the term as it is a nontechnical descriptor.
Xenobot	Kriegman	Synonymous with reconfigurable organisms.
Xenobot	Bongard	A particular set of trajectories through frog attractor space.
Xenobot	Levin	A proto-organism formed as a default by frog ectodermal cells deprived of normal instructive influences by the other frog embryo cells.
Biomachine	Blackiston	Preferred term for the systems created by this team. Applied when behaviors are used or designed for a prespecified purpose and falls under the umbrella of multicellular-engineered living systems.
Biomachine	Levin	A construct containing at least some living, naturally evolved components but which is configured so as to afford a degree of rational control over its form and function, toward some useful purpose, by another agent (e.g., an engineer or worker in regenerative medicine).
Agential materials	Levin	A material for which optimal engineering protocols require behavior shaping (not just micromanagement) because the components are not passive but have some degree of autonomous behavior, preferred states, and decision-making capacity in some problem space.
Reconfigurable organism	Team	A living system reconfigured manually or automatically for a prespecified purpose.
Computer-designed organism	Kriegman, Bongard, Levin	A public-facing term to distinguish manually designed biomachines from automatically designed ones.
Computer-designed organism	Blackiston	A public-facing term to distinguish manually designed biomachines from automatically designed ones but would not characterize the biomachines as organisms. Would use computer-designed living system.

This raises a tricky question: At what point do living tissues become a robot? Indeed, there are many different pieces of a developing animal that, when isolated from the host, can move and sense on their own. Parts of animals are not robots. However, once we artificially combine and shape them to render desired behaviors, they are no longer merely animal parts, they become artifacts: artificial yet fully biological robots.

If removed from early frog embryos, a small piece of ectoderm called the animal cap will mature to form a ball of skin covered in motile cilia, which can propel the ball forward through water. Early tadpoles glide around the same way, at the same speed as this explanted tissue. However, there are several ways to deflect (or “program”) the system away from its natural behavior. In our work to date, we have shaped these tissues into quadrupeds, bipeds, pyramids, toruses, semi-toroids, and various other nonspherical body shapes (Fig. 1). In some designs, we removed cilia entirely and relied instead on localized patches of cardiac tissue to produce volumetric actuation. Different body shapes, and different admixtures of tissue types, resulted in different (and thus artificial) behaviors.

The resulting “xenobots” are *autonomous*: they are able to maintain their structure (and thus function) without human intervention and exhibit a diverse array of behavioral repertoires. They are also *adaptive*: xenobots can repair their structure after significant damage. But are they *intelligent*?

The xenobots reported to date have no known mechanism of sensor–motor coordination. All the cells within a xenobot can sense and act and talk to their neighbors, but xenobots as a whole do not exhibit perceptive (sense-guided) behavior: Their movement is consistent with models of blindly actuating bundles of motors (open-loop control). Their healing is consistent with models of blindly adhering bundles of sticky particles. Sometimes xenobots appear to behave perceptively: They suddenly “decide” to turn around, or they become “interested” in an object and “examine” it repeatedly. However, the very same behaviors are manifested by quasi-stable bundles of motors when tipped into a new orientation.^47^ This is the problem of ascribing a capacity to an agent solely on the basis of its behavior. But this does not preclude future xenobots from genetic modifications that give rise to sense-guided behavior, action alternatives, memory, learning, and other increasingly cognitive behaviors.

Formally, we have referred to xenobots as reconfigurable organisms, a nod to reconfigurable modular robots^48,49^ in which robot modules (like the cells within a frog embryo) can be attached, detached, and rearranged to form new structures (configurations). As biorobotics researchers begin to reconfigure other organisms, the term “xenobot” might be reserved to describe a particular subset of reconfigurable organisms. If biological robots are one day built out of cells harvested from the African elephant (*Loxodonta*) instead of the African clawed frog (*Xenopus laevis*), then perhaps they will be called “loxobots” instead of “xenobots.”

(This is not to say the choice of “frog modules” was entirely arbitrary. Developing frog eggs, and xenobots derived from them, survive in fresh water without food; mammalian-derived robots such as loxobots would require special media with growth factors and nutrients and precisely tuned CO_2_ levels.) There is also the possibility of chimeras (mixtures of species) and biohybrids (mixtures of living and artificial materials). Any one of the creatures in this menagerie is just as likely to invoke the literal interpretation of xenobots: “strange robots.”

Whether we call them robots or organisms, and whether or not they become chimeras or cyborgs, designing and optimizing such systems is not only strange but also extremely nonintuitive. To make the design problem tractable for human minds, the solution space must be winnowed down to a vanishingly small subset of possible forms and functions. Breaking free from design constraints imposed by human cognitive limits would greatly widen our search for useful technologies and new knowledge, but it will by definition require nonhuman assistance. Computational tools (old^8,50–54^ and new^9,55–57^) are poised to help.

**FIG. 2.**
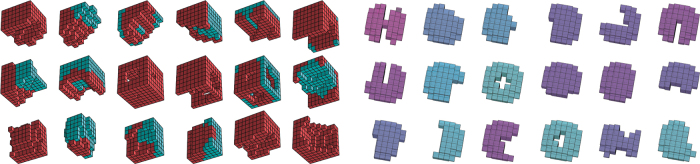
Sample xenobot designs optimized by machine learning methods. *Left*: xenobots designed for locomotion using myocardial tissue (*red*). With permission from ref. [8]. *Right*: xenobots designed for kinematic self-replication. With permission from ref. [9].

We used computers to rapidly generate a diversity of buildable xenobot forms that maximized a desired behavior and maintained that behavior across a range of simulated conditions (Fig. 2). This led us into parts of design space human engineers typically do not wander. There, we not only found creative xenobot designs (fractals, strange asymmetries, and porous structures) but also new biological design principles (e.g., how the right morphology can cohere the noisy actions of its parts).

Exploring this space in simulation allowed us to filter out billions of bad designs before attempting to build them in reality. This greatly improved efficiency in the wetlab and reduced biowaste. But this process can be energy-intensive and its carbon footprint grows with each second of simulation time. This is arguably the most pressing ethical concern of xenobot research. Future work should thus strive to encode xenobot design space such that, instead of meandering through random design variants,^58^ gradients can be efficiently followed toward optimal solutions.^59^ If successful, this move from trial-and-error to gradient descent could reduce processing time, and thus carbon footprint, by several orders of magnitude.

Biological robots, however efficiently designed, may never become a viable technology. This fact can get lost in the rhetoric surrounding xenobots. When engaging with the popular press and funding agencies (or when trying to explain your research to your grandma), it is helpful to articulate a compelling purpose. We have communicated several visions of the future in which xenobots clean up the environment, monitor industrial processes, deliver medicine, and even fight cancer. It is important to remember that xenobots are not yet useful: they cannot solve any problems at all.

This does not mean that they are useless. Biological robots, like many of the soft robots reported in this journal, challenge conventional views of robotics. They force us to think creatively about how to achieve even the most basic behaviors. The knowledge we gain from our attempts to optimize their design, formalize their control, broaden their functionality, and enhance their intelligence, will eventually become embodied in future useful technologies, whether they are composed of cells, steel, or silicone.

## Expanding Robotics by Combating Dichotomous Thinking (Bongard Commentary)

Like every other endeavor, science is not exempt from the limits of human cognition. Primary among these limits is dichotomous thinking: Making sense of nature, and channeling what we know into embodied machines or disembodied neural networks, is easier if we impose distinctions on natural phenomena: for example, organisms have separable bodies and brains, and sense organs and actuation systems, and brain regions, and so on. However, nature need not, and does not respect human attempts to draw boundaries. What follows are some of the most obvious distinctions the scientific community has attempted to impose on nature, and how technological and biological advances are increasingly corroding them.

In genetics, a clear separation between genotype and phenotype has been imposed ever since genes were first hypothesized and then discovered. However, advances in epigenetics and synthetic biology are increasingly revealing that genes, environment, and phenotype are often more usefully thought of as a coupled dynamical system. Xenobots, for instance, support the idea that a developing organism represents a trajectory through an attractor space. The “default phenotype” usually arrived at in response to environmental signals experienced by the organism in its natural environment is but one attractor in this space. Environmental change, ectopic perturbation, or even AI-designed tissue rearrangements can push a collection of cells into stable adult forms completely different from the one usually observed in nature.

In robotics and AI, Cartesian dualism—the West's most famous exemplar of dichotomous thinking—has biased the kinds of scientific questions we ask about intelligence, and the kinds of engineering approaches we take in attempting to create artificial intelligence. Indeed, the very bicameral nature of the robotics and AI communities illustrates how Cartesian dualism warps the field(s). Evidence is now mounting that Cartesian dualism has driven AI into a local optimum: state-of-the-art non-embodied AI is just as vulnerable to adversarial attacks or out-of-distribution environments as it always was,^60^ and calls to incorporate causal reasoning into AI are growing^61^ because non-embodied deep networks cannot cause effects and reason about the results.

Our self-replicating xenobots upend a third form of dichotomous thinking that is poisoning computational research: the distinction between tape and machine. This distinction goes all the way back to Turing's original formulation of a theoretical nonhuman “computer.” However, there is no clear “tape” and “machine” in our self-replicating xenobots: nowhere is there some formal instruction to “find loose cells and push them into copies of yourself.” Indeed the xenobots' ability to self-replicate emerges from the collapse of all three forms of dichotomous thinking mentioned above. First, the form and function of the xenobots arise from complex feedback loops between their “genotype” (genetically unmodified frog DNA), phenotype (shape and movement pattern), interoceptive environment (cells and their neighbors within a single xenobot), and exteroceptive environment (the replicative raw material of dissociated cells, and other xenobots).

Second, the “body” of a xenobot is not a binary property during self-replication: there are simply smaller and larger piles of frog cells, and less- or more-motile piles. And, a xenobot's “brain” is the net result of aneural intercellular electric, mechanical, and chemical communication. (Thus, also providing another challenge to Cartesian brain/body distinction by reminding us that there is a diversity of intercellular architectures that can collectively drive increasingly complex behavior.) Third, the geometry of each xenobot dictates how it moves and how, or whether, it contributes to replication: in other words, “the shape is the tape.”

Xenobots are thus an ideal guide for leading us, step by step, into the increasingly deep waters of total morphospace. We must leave behind the shallows, where animals and robots with distinct bodies and brains, genotypes and phenotypes, tapes and machines, swim. Such agents do not actually exist. They only exist in our imaginations because they are the easiest kinds of agents for us to understand. We must leave them and dichotomous thinking behind, and instead learn to swim in the deeps, where real animals reside, and where really intelligent machines will reside. Such animals and machines are ever-shifting patterns of intricately interdependent and fractally arranged bodies and brains, formal descriptors, and physical structures, made that way by the ever shifting currents of natural and artificial selection: it is admixture, all the way down. Such creatures may yield to our understanding, but they will not yield to our attempts to divide them.

## Expanding Biology: What Biorobots Tell Us About Evolution, Morphogenesis, and Control (Levin Commentary)

Developmental biology began as a study of the phenomenology of specific embryonic model systems. Modern developmental biology, however, comprises not only embryogenesis but also regeneration in adult organisms, metamorphosis, and increasingly—digital and synthetic morphogenetic systems.^62–65^ What binds these diverse biological examples together into a field is the ability to go beyond specific instances toward questions of multiscale control: How do single cells (which used to be organisms themselves) cooperate toward invariant form and function?^66^ How does this process increase complexity while resisting external perturbations and harnessing noise, and how does evolution give rise to self-assembling complex systems with both built-in and learned behaviors?

Exclusive focus on the *N* = 1 example of life provided by Earth's specific phylogenetic tree, with its baggage of frozen accidents, obscures deep principles of life-as-it-can-be.^67^ Testing our theories with data outside the data set of forms that generated them is an essential aspect of any science, crucial not only for possible exobiology but also to gain a deeper understanding of evolvability, plasticity, and the relationship between genomically specified cellular hardware and the physiological software of life. Understanding the rules of life (moving from Earthly zoology/botany to a fuller option space of possible living things) requires us to recreate and analyze novel living constructs never-before existing on Earth.

Thus, the study of bioengineered forms and their multiscale control policies are an important part of a maturing developmental biology. Xenobots are a good example in which to examine the converse blurring of lines between classical robots and organisms. They challenge us to ask key questions about what we mean by “robot” and “machine,” and whether those binary categories really facilitate understanding and progress.^68,69^ They challenge us to expand traditional notions of a “program” beyond linear code written by a human to probabilistic, parallel, naturally evolving control policies embodied in biological components that enable the highly flexible, adaptive interoperability of life seen in chimeras and biohybrid constructs.^70^

Cells are routinely thought of as molecular machines by cell biologists and bioengineers tasked with modifying their behavior;^71,72^ one of the key challenges of the coming decades is to develop frameworks (or borrow them from information science, cybernetics, computer science, and behavior science) with which to understand the principles by which these remarkable, plastic, evolved machines robustly scale to solve morphogenetic and physiological problems. Developmental and regenerative plasticity^73^ clearly reveal that multicellular collectives can handle large degrees of novelty; how does evolution result in robust plasticity, and how can we take advantage of these architectures for improved robotics and artificial intelligence? Xenobots reveal new ways to think about control, design, and the multiscale behavior across the spectrum of natural and artificial systems.^74–76^

Are xenobots engineered or natural products of what *Xenopus* cells do already? Yes, both. Engineering is not just about adding new ingredients, such as DNA plasmids for synthetic biology circuits or nanomaterials (although these will certainly be added in the future). Xenobots reveal a new strategy for the bioengineer's toolbox: programming desired form and function by releasing natural constraints. In xenobots, nothing was added; instead, cells were liberated from the normal constraints of the rest of the embryo. This led to the discovery that cells' baseline capability is not merely to form a boundary that keeps pathogens out of the organism. Instead, this mundane two-dimensional lifestyle is forced on them by the instructive interactions of other cell types. When allowed to express their own multicellularity, these epithelial cells do not die, form a monolayer, or wander aimlessly. Instead, their baseline behavior is to form a self-motile proto-organism with individual and collective behaviors, such as kinematic self-replication.^9^

These types of form and function were not obvious from any first principles or from their wild-type genotype and illustrate the need to understand what it is that the evolutionary process taught the *X. laevis* genome;^77,78^ it was not only how to make a frog or a tadpole. There has never been selection to form a functional xenobot, which raises the important question of what defines the class of constructs that cells can make, beyond the default configuration supported by eons of selection forces. What else do these, and all the other, cells know how to do, in novel circumstances? Of course, their capabilities are explainable, after their discovery, as genetically specified features (such as cilia and adhesion proteins) working in novel ways through the laws of physics.

We encourage readers to make (and register in advance) predictions of other capabilities that cells such as these will exhibit—the degree of their plasticity and the specific forms and behaviors of which they might be capable. The predictions of their capacities and limitations (so-called developmental constraints) will be interesting to test against the rapidly emerging data in this field. The fact that these are no more predictable *ab initio* from genomic information and current models than the primary shapes of animals and plants is an important challenge to the field.

The xenobots' ability to assemble the next generation from cellular material in their environment sheds an important light on the notion of control, both in the evolutionary and in the engineering context. Indeed, both use the same strategy: reliance on the competency of their components. Engineers make the first generation of xenobots by relying on the willingness of cells to get together despite novel circumstances and create a motile coherent agent. These xenobots create their next generation by exactly the same process: they assemble the cells and impact the size and composition of the collective, but then take advantage of the competency of cells to do the rest—guided self-assembly (i.e., behavior shaping), not micromanagement is the way that we, and the xenobots themselves, make more xenobots. Indeed, evolution itself relies on the competency of cells, tissues, and organs to solve problems.^79^

**FIG. 3.**
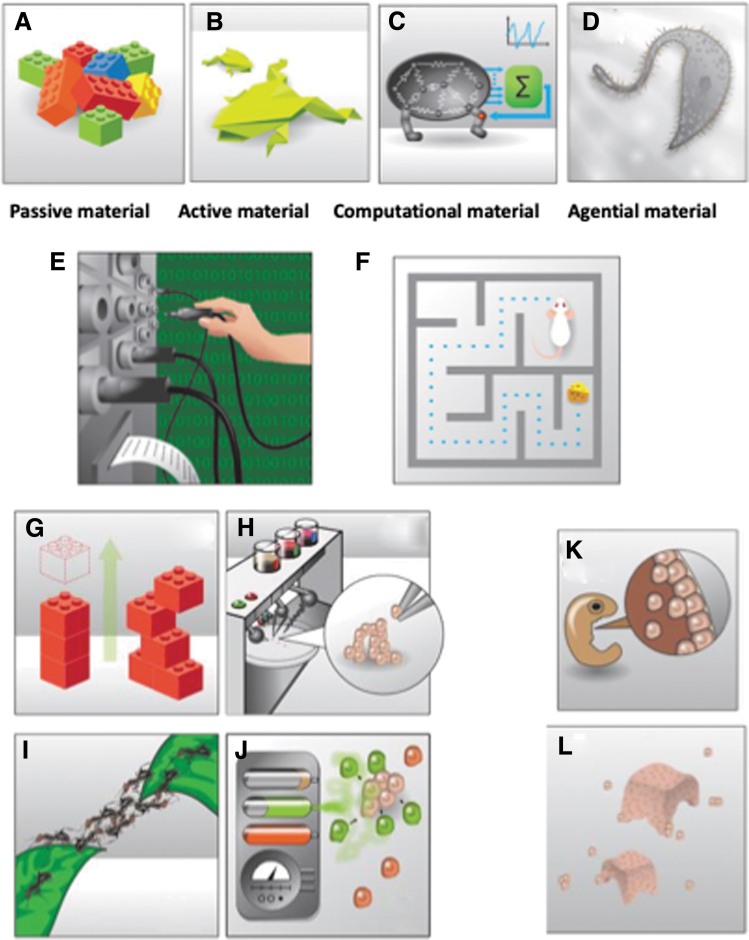
Engineering with agential materials. Engineering has traditionally been carried out with passive materials **(A)**, which can only be dependent on to keep their shape and other physical properties. These must be carefully managed for each desired functionality, giving rise to a perception of robotics as the manual arrangement of parts toward each goal. However, increasingly, engineering has moved toward active matter **(B)** and computational media **(C)** as well recognized in soft robotics;^79–81^ now, biorobotics enters a new phase transition where the material is “agential”—it is composed of subunits **(D**, living cells**)** which themselves were whole organisms once and thus have many built-in competencies and agendas,^82^ including problem-solving in physiological, metabolic, and morphological problem spaces.^78,79,83,84^ This means that robots are now not only constructed by physical (or even genetic) rewiring **(E)** but more akin to behavior-shaping **(F)**, using signals and environments to achieve desired system-level behavior. In contrast and complement to 3D-printing and similar approaches designed for building with passive matter **(G)**, which also works with cells **(H)**, collective intelligence of living systems at all scales (such as that of an ant swarm, **I)** can be used to manipulate the collective behavior of cells **(J)** in anatomical morphospace, by signaling that alters the collective's navigation policy of that space: just as instructive signals from other cells cause frog ectodermal cells to be a two-dimensional barrier in standard embryos **(K)**, techniques such as subtraction (of other cells and their signals) and stimuli can achieve guided self-assembly toward novel form and function **(L)**. 3D, three-dimensional. All images by Jeremy Guay of Peregrine Creative, used with permission.

Making a copy of a biobot does not have to be micromanaged any more than the original biobot needed to be three-dimensional (3D) printed. This does not resemble traditional robotics because the history of engineering has relied on *passive* materials. Progress is revealing a continuum of control strategies appropriate to active, computational, and *agential materials* (Fig. 3): building things with parts that have context-sensitive activity, computational capacity, and behavioral agendas that optimize various parameters in local problem spaces. Evolution likewise relies on its components to provide more than reliable passive form (LEGOs that hold their shape but do nothing else)—instead, it largely shapes signals and biophysically implemented incentives to modulate the behavior of cells, tissues, and organs.

Biologists, roboticists, and intellectual property lawyers will have to become comfortable with workflows in which we are literally collaborators with our artifacts, parts of which will actively build the next level of the construct in ways that are not fully captured by a craftsman's action protocol. This has massive advantages, for rational design and for evolution, because it allows both to work in a simpler space of incentives and inputs, not microstates. It also provides extensive challenges because our science of prediction and control of systems with a multiscale competency architecture is still nascent.

The existing variants of xenobots have not yet been provided with edited genomes, new materials, and so on. Counter to established intuitions, the kind of control expected of robotic platforms (machines) does not require genomic editing. Computer science achieved the information technology revolution by moving from programming by hardware rewiring to software control via inputs. Evolution discovered this trick via physiological software very early on,^85^ and recent advances in the control of growth and form show how much anatomical change can be implemented by interventions that do not change the underlying hardware (genetics).^86^

Induction of appendage regeneration, production of structures belonging to other species despite a wild-type genome, induction or normalization of cancer, and so on, can all be induced by biophysical information signals and often invisible to canonical molecular biology tools.^87^ A key aspect of the early generations of xenobots is precisely that they reveal the plasticity of cells' ability to explore morphospace and behavioral space with the same genome. This is an expansion of “epigenetics,” toward a better understanding of plasticity and the actual products of evolution.

The practical applications of such biobots are numerous. Major advantages are their biodegradable, soft nature, and the built-in competencies of cells for sensing, metabolic/biochemical activity, and actuation in ways that are far beyond today's engineering efforts. Most obvious are the useful living machines, which could microsculpt bioengineered tissues *in vitro*, perform sensing or cleanup in many industrial processes, environmental cleanup, or even in-body biomedical applications with respect to injury, infectious microbes, and cancer. They also provide an inexpensive safe model system for education in STEM efforts (such as Frugal Science).

Deeper impacts include the use of this platform as a sandbox in which to crack the morphogenetic code and move closer to the endgame of an “anatomical compiler.” Work in biobots will help address the gaps in our ability to predict and control complex anatomical shapes^66,88^ for applications in developing signaling protocols to induce cells in the body to achieve correct regenerative morphogenesis. An essential aspect of such strategies to address birth defects, traumatic injury, and errors of multicellularity such as cancer and aging is learning to control the shape toward which cellular collectives cooperate. Biobots are an enabling platform to achieve Feynman's dictum—to truly understand morphogenetic systems by building them ourselves.

Benefits will also accrue to computer science and robotics in inorganic media because the fascinating features of biobots do not derive from any magic of protoplasm. Rather, they derive from their multiscale competency architecture and other principles to be discovered. Current robotics is safe from cancer (defections of components from system-level goals) but is brittle and lacking general intelligence. The opportunity is to borrow from nature, not the specific genes and pathways comprising today's developmental biology textbooks, but the ways in which non-neural systems solve problems in diverse problem spaces by scaling their basal homeostatic properties into larger goals.^89,90^ The implications for AI and behavioral science in general concern biobots' degrees of proto-cognitive sophistication. No claims have yet been made for the degree of agency they can exhibit (this characterization is currently under way), but it is clear that humans' intuitions about recognizing intelligent behavior are highly limited by the training set of familiar animals behaving in 3D space.^79,91^

The many surprises in the basal cognition literature^92^ reveal that we are fundamentally not good at recognizing possible intelligence in navigating physiological, morphological, and other non-obvious spaces; a rich research program exists around efforts to understand xenobots' unconventional intelligence (at the very least, as a warm-up to exobiological and AI advances). Finally, the long-term implications of this research program address a key component of avoiding existential risk for humanity: better learning to understand and predict the behaviors of systems composed of competent components (emergent properties of collective intelligences).

This technology also has important implications for ethics.^93,94^ These are not limited to familiar biosafety concerns, as these technologies are much easier to contain and have much better safety profiles than existing efforts in synthetic bacteria, engineered viruses, and genetically modified crops and other organisms. Xenobots, for example, live only in very specific environments and are highly dependent on humans for their manufacture; they biodegrade rapidly—unlike many of today's synthetic biology efforts that replicate readily in the biosphere. An aspect often forgotten is that we cannot just evaluate potential *risk* as if the status quo was excellent, and our goal was merely not to make things worse. Human and animal suffering worldwide is enormous and due to a poor understanding of system-level biological control.

There is a massive opportunity cost of not pursuing this research because of its potential to advance regenerative medicine and improve quality of life worldwide. Any risk calculations of such technologies must balance against the moral duty to pursue scientific programs that can alleviate unmet environmental and biomedical needs and health care disparities. We ethically owe it to victims of birth defects, injury, cancer, and degenerative disease, and so on, and to the animal model systems currently used for biomedical research (which could be partially replaced by simplified constructs), to better understand how to influence cells to achieve complex structural and functional states.^95,96^ Another important aspect of ethics in this kind of work is that it forces us to confront rules based superficial criteria—past distinctions of origin and composition which at one point might have been a reasonable guide to how one ought to treat a given system but are not fundamental and based only on past limitations of technology and imagination.

Human society has a long history of moral failures toward numerous types of human and nonhuman animals based on distinctions that are now widely recognized to be irrelevant. By forcing us to search for deeper essential meanings of categories that drive moral concern and responsibility (agency, intelligence, etc.) in unfamiliar novel guises, we will be better placed to more appropriately implement compassion in our relationship to a truly diverse community of beings.

## Conclusions

Robotics is an attempt to engineer useful, partly or fully autonomous artifacts that remind us in some way of organisms. Increasingly, this research is breaking with the classical conception of a robot as necessarily made of passive inorganic components and made or designed piece-by-piece by humans at every step. We believe that the essential nature of robotics is not limited to a specific kind of material, origin story, or type of control. However, this stance necessitates a re-evaluation of terminology as multidisciplinary teams bring field-specific techniques to bear on new questions. As we lay out, this terminology remains in a state of flux, as audiences continue to view these questions through their own unique lens.

Recent work in biohybrid constructs—machines made of cells^3,34,35,75,97–107^—is giving rise to a very dynamic emerging field. We do not here comprehensively review all the existing work (see Ebrahimkhani and Levin,^65^ Kamm and Bashir,^72^ and Kamm et al^108^), but rather to provide our individual perspectives on specific issues central to a number of communities in the biological and engineering sciences. Nor are we attempting to review the growing literature on robot design automation,^58,109,110^ which has clearly shaped our own contributions and perspectives and is constantly evolving.

We suggest that many new research programs challenge the traditional lines drawn between a machine, a robot, and an organism. Living, “active,” “smart,” or “agential” materials (depending on the author) have much to offer robots and engineering disciplines. Simultaneously, materials science research can provide insights into basic developmental biology and bioengineering as cells and tissues assemble during the design process. From the opposite end, simulation and AI-driven design have the potential to enhance traditional biology programs, as sim-to-real approaches continue to accelerate *in vivo* and *in vitro* discovery.

Within the group, the authors vary in specific terminology and system label usage (e.g., robot vs. organism vs. xenobot), whether they characterize designs as a group of skin cells or something new, and when speculating about the degree of sensory motor communication possible in the system. These issues remain areas of active investigation. Finally, we understand where disagreements arise among fields and hope the views presented here help frame the research program to the larger scientific community.

Many fields of science and technology, as they mature, go beyond the specific matter of their early applications. Engineering is no longer limited to the study of engines. Just as the deep principles of physics and thermodynamics apply across scales, from galaxies to the soft matter of cellular processes, biology is moving from zoology to unraveling the fundamental laws of life across natural, synthetic, and exobiological systems. Computer science and robotics can lead the way, with their early emphasis on functionality and realizability, not material implementation.

Thus, importing the ideas of programmability and substrate independence will open further opportunities in the life sciences and bioengineering, to go beyond the contingent natural products of evolution to truly understand life in diverse configurations. Together, the emerging consilience of techniques and concepts at the intersection of materials science, computing, and bioengineering is an exciting new field with the potential to create technological machines that embody biological principles, and incorporate biological components, in entirely new ways.

## Author Biographies

**Douglas Blackiston** has a BSc in Biological Sciences from McDaniel College and a PhD in Biology from Georgetown University. His research examines the relationship between organism-level behaviors and developmental events, including the ability of memory to persist through metamorphosis in holometabolous insects and the sensory–motor integration of transplanted eyes in vertebrate models. His interest in biological machines and robots arises from this background, applying developmental and behavioral techniques from model organisms to engineer multicellular living systems with behaviors designed for specific purposes.

**Sam Kriegman** is an assistant professor of computer science, chemical and biological engineering, and mechanical engineering at Northwestern University. His research draws inspiration from the origin and evolution of life and applies the underlying mechanisms of self-organization and natural selection to the creation of novel autonomous machines. These machines can in some cases perform useful work, or they may be used as scientific tools to understand how animals evolve, grow, move, sense, and think.

**Josh Bongard** is the Cyril G. Veinott professor of computer science at the University of Vermont. His laboratory is dedicated to understanding how embodied cognition manifests in living systems, and how such discoveries can open new pathways to creating intelligent, safe, and understandable algorithms and machines. Such technologies can then, in turn, suggest new hypotheses about how organisms continuously transform themselves to track and exploit opportunities presented by their internal and external environments.

**Michael Levin** is distinguished professor of Biology at Tufts University and an associate faculty at Harvard's Wyss Institute for Biologically Inspired Engineering. He has dual undergraduate degrees in computer science and biology and a PhD in Genetics from Harvard Medical School. He has a long-time interest in artificial life and emergent morphogenetic and behavioral capacities in natural and artificial multiscale agential systems. His laboratory uses developmental biophysics, computational modeling, and behavioral science to understand morphogenesis and develop applications across birth defects, regenerative medicine, and cancer.
